# An omnibus permutation test on ensembles of two-locus analyses can detect pure epistasis and genetic heterogeneity in genome-wide association studies

**DOI:** 10.1186/2193-1801-2-230

**Published:** 2013-05-19

**Authors:** Damrongrit Setsirichok, Phuwadej Tienboon, Nattapong Jaroonruang, Somkit Kittichaijaroen, Waranyu Wongseree, Theera Piroonratana, Touchpong Usavanarong, Chanin Limwongse, Chatchawit Aporntewan, Marong Phadoongsidhi, Nachol Chaiyaratana

**Affiliations:** 1 Department of Electrical and Computer Engineering, Faculty of Engineering, King Mongkut’s University of Technology North Bangkok, 1518 Pracharat Sai 1 Road, Bangsue, Bangkok 10800, Thailand; 2 Department of Computer Engineering, Faculty of Engineering, King Mongkut’s University of Technology Thonburi, 126 Pracha-utid Road, Bangmod, Toongkru, Bangkok 10140, Thailand; 3 Division of Technology of Information System Management, Faculty of Engineering, Mahidol University, 25/25 Phuttamonthon 4 Road, Nakhon Pathom 73170, Salaya, Thailand; 4 Division of Molecular Genetics, Department of Research and Development, Faculty of Medicine Siriraj Hospital, Mahidol University, 2 Prannok Road, Bangkok 10700, Bangkoknoi, Thailand; 5 Department of Mathematics and Computer Science, Faculty of Science, Chulalongkorn University, 254 Phayathai Road, Pathumwan, Bangkok 10330, Thailand

**Keywords:** Attribute selection, Complex disease, Epistasis, Genetic heterogeneity, Genome-wide association study, Pattern recognition, Permutation test, Single nucleotide polymorphism, Type 1 diabetes mellitus

## Abstract

This article presents the ability of an omnibus permutation test on ensembles of two-locus analyses (2LOmb) to detect pure epistasis in the presence of genetic heterogeneity. The performance of 2LOmb is evaluated in various simulation scenarios covering two independent causes of complex disease where each cause is governed by a purely epistatic interaction. Different scenarios are set up by varying the number of available single nucleotide polymorphisms (SNPs) in data, number of causative SNPs and ratio of case samples from two affected groups. The simulation results indicate that 2LOmb outperforms multifactor dimensionality reduction (MDR) and random forest (RF) techniques in terms of a low number of output SNPs and a high number of correctly-identified causative SNPs. Moreover, 2LOmb is capable of identifying the number of independent interactions in tractable computational time and can be used in genome-wide association studies. 2LOmb is subsequently applied to a type 1 diabetes mellitus (T1D) data set, which is collected from a UK population by the Wellcome Trust Case Control Consortium (WTCCC). After screening for SNPs that locate within or near genes and exhibit no marginal single-locus effects, the T1D data set is reduced to 95,991 SNPs from 12,146 genes. The 2LOmb search in the reduced T1D data set reveals that 12 SNPs, which can be divided into two independent sets, are associated with the disease. The first SNP set consists of three SNPs from *MUC21* (mucin 21, cell surface associated), three SNPs from *MUC22* (mucin 22), two SNPs from *PSORS1C1* (psoriasis susceptibility 1 candidate 1) and one SNP from *TCF19* (transcription factor 19). A four-locus interaction between these four genes is also detected. The second SNP set consists of three SNPs from *ATAD1* (ATPase family, AAA domain containing 1). Overall, the findings indicate the detection of pure epistasis in the presence of genetic heterogeneity and provide an alternative explanation for the aetiology of T1D in the UK population.

## Background

Epistasis or gene-gene interactions are among many causes of complex diseases (Moore 2005). In the simplest form, epistasis can be described by two-locus disease models, in which both loci jointly contribute towards the disease susceptibility (Neuman and Rice 1992; Schork et al. 1993). Many attempts have been made to provide consistent definitions of epistasis (Cordell 2002; Hallgrímsdóttir and Yuster 2008; Li and Reich 2000; Marchini et al. 2005; Musani et al. 2007; Verhoeven et al. 2010). Regardless of preferred definitions, a common ground for describing epistasis covers an effect deviating from the combined individual effects of each genetic factor. In other words, epistasis describes an effect that departs from a linear addition of individual effects (Fisher 1918). The detection of epistasis hence provides necessary information complementary to that gained through single-locus analysis.

With the availability of genome-wide genotyping technologies, a large number of single nucleotide polymorphisms (SNPs) can be considered during epistasis detection (Heidema et al. 2006; Motsinger et al. 2007; Van Steen 2012). At present, the most feasible strategy for genome-wide epistasis detection involves two-locus analysis (Evans et al. 2006; Gayán et al. 2008; Ionita and Man 2006; Liu et al. 2011; Marchini et al. 2005; Sha et al. 2009; Wongseree et al. 2009). The detection may concentrate on all possible SNP pairs (Gayán et al. 2008; Liu et al. 2011; Marchini et al. 2005; Wongseree et al. 2009) or only SNP pairs where at least one SNP in each pair exhibits a marginal single-locus effect (Evans et al. 2006; Ionita and Man 2006; Liu et al. 2011; Marchini et al. 2005; Sha et al. 2009). Exhaustive two-locus analysis is generally required when pure epistasis (Culverhouse et al. 2002) is present. This is because each interacting SNP in a purely epistatic model exhibits no marginal single-locus effect. Although the importance of pure epistasis remains in question (Cordell 2009), many genetic association studies reveal that putatively pure epistasis plays a role in determining disease susceptibility (Cho et al. 2004; Jiang and Neapolitan 2012; Zhang et al. 2008).

In addition to epistasis, two-locus (Hallgrímsdóttir and Yuster 2008; Li and Reich 2000; Neuman and Rice 1992; Schork et al. 1993) and multi-locus disease models (Edwards et al. 2009; Lunetta et al. 2004) also describe other phenomena. One particular phenomenon that makes the capture of genetic factors responsible for complex diseases a difficult task is genetic heterogeneity. Basically, genetic heterogeneity models define independent effects that cause the same complex disease. Since it is impossible to know beforehand that each affected individual participating in genetic association studies is predisposed to which independent effect, the presence of genetic heterogeneity always leads to the reduction in statistical power to detect causative SNPs (Edwards et al. 2009; Lunetta et al. 2004; Meng et al. 2009; Ritchie et al. 2007; Ritchie et al. 2003).

From a machine learning viewpoint, the identification of causative SNPs among available SNPs in genetic association studies can be treated as an attribute selection problem. The aim of attribute selection is to identify informative attributes necessary for the correct classification of recruited samples. Saeys et al. (2007) categorise attribute selection techniques into three main approaches: filter, wrapper and embedded approaches. The filter approach interests in identifying SNPs associated with the disease according to a statistical or mathematical measure. The wrapper approach attempts to search for the best SNP combination that provides the highest prediction accuracy dictated by a classifier. The embedded approach uses available SNPs to construct a prediction model while simultaneously prioritises informative SNPs.

Among the wrapper techniques, a technique which is proven to be capable of detecting pure epistasis in the presence of genetic heterogeneity is a multifactor dimensionality reduction (MDR) technique (Edwards et al. 2009; Ritchie et al. 2007; Ritchie et al. 2003). MDR searches for the best SNP combination that yields the highest prediction accuracy according to the rules governed by multi-dimensional decision tables (Ritchie et al. 2001). Although the detection power of MDR is high, the demonstration has been limited to simulations consisting of two independent purely epistatic two-locus interactions. Moreover, MDR is a time-consuming technique and hence requires large computational efforts for multi-locus analysis in genetic association studies with a large number of SNPs (Edwards et al. 2009; Kwon et al. 2012; Pattin and Moore 2008; Ritchie et al. 2001; Wongseree et al. 2009).

Similar to MDR, a random forest (RF) is an embedded technique which is also proven to be capable of detecting epistasis in the presence of genetic heterogeneity (Lunetta et al. 2004; Meng et al. 2009). RF consists of multiple decision trees in which each tree is randomly constructed from available SNPs. Causative SNPs can be identified by permuting the genotype of each SNP and observing how this affects the overall prediction accuracy (Breiman 2001). The detection power of RF has been demonstrated through simulations involving multiple independent epistatic multi-locus interactions. Nonetheless, the previous studies concentrate on epistasis with marginal single-locus effects. As a result, the ability of RF to detect pure epistasis has not yet been determined.

Unlike genetic association studies that use wrapper and embedded techniques, most studies involving filter techniques rarely consider scenarios which cover genetic heterogeneity. However, one filter technique which should be suitable for detecting pure epistasis in the presence of genetic heterogeneity is an omnibus permutation test on ensembles of two-locus analyses or 2LOmb (Wongseree et al. 2009). 2LOmb exhaustively performs two-locus analysis on case-control SNP data by *χ*^2^ tests. The best ensemble of SNP pairs is then progressively constructed where the statistical significance of the association between the ensemble and the disease is determined by a permutation test. 2LOmb is suitable for detecting purely epistatic two-locus interactions and purely epistatic multi-locus interactions with marginal two-locus effects (Wongseree et al. 2009). In addition, 2LOmb has been successfully benchmarked against an exhaustive two-locus analysis technique, a set association approach (Hoh et al. 2001), a correlation-based feature selection technique (Hall and Holmes 2003) and a tuned ReliefF technique (Moore and White 2007). Although the study has been conducted without considering genetic heterogeneity, the result from an application of 2LOmb to a real case-control data set, derived from a genome-wide data set by focusing on SNPs within or near candidate genes, suggests that 2LOmb can function when genetic heterogeneity is present. Previously, 2LOmb identifies 11 intronic SNPs which exhibit no marginal single-locus effects and are associated with type 2 diabetes mellitus (T2D) in a UK population (The Wellcome Trust Case Control Consortium 2007): four SNPs in *PGM1* (phosphoglucomutase 1), two SNPs in *LMX1A* (LIM homeobox transcription factor 1, alpha), two SNPs in *PARK2* (parkinson protein 2, E3 ubiquitin protein ligase (parkin)) and three SNPs in *GYS2* (glycogen synthase 2 (liver)). The results also suggest that there are no interactions between genes (Wongseree et al. 2009). Obviously, this finding signifies the power of 2LOmb to detect genetic heterogeneity. Nevertheless, a thorough investigation by simulations is still required. In addition, the possibility of applying 2LOmb to a genome-wide data set also needs to be explored.

In this article, the ability of 2LOmb to detect pure epistasis in the presence of genetic heterogeneity is demonstrated. 2LOmb is benchmarked against MDR and RF in various simulation scenarios generated by varying the number of available SNPs, number of causative SNPs and ratio of case samples in which the disease status is governed by different purely epistatic interaction models. The statistical power of 2LOmb to directly identify the number of independent interactions in simulated data from its output is subsequently evaluated. The application of 2LOmb to a genome-wide type 1 diabetes mellitus (T1D) data set is also included. In this study, the genome-wide T1D data set is chosen instead of the T2D data set because 2LOmb does not detect any purely epistatic interactions in the T2D data set.

## Results and discussion

### Testing with small-scaled simulated data

2LOmb is benchmarked against MDR and RF in a simulation trial involving both pure epistasis and genetic heterogeneity. An output from an efficient algorithm should contain a low number of SNPs and a high number of correctly-identified causative SNPs. These two measures on the number of SNPs are the performance indicators. Each simulated data set contains 20 or 1,000 unlinked SNPs in which two independent purely epistatic interactions are present. Each interaction is based on one of the models investigated by Wongseree et al. (2009) and is governed by two, three or four causative SNPs. As a result, the interesting numbers of causative SNPs in each data set are 4 (2&2), 5 (2&3), 6 (3&3 or 2&4), 7 (3&4) and 8 (4&4). The allele frequencies of all causative SNPs are 0.5; these are dictated by the purely epistatic models with penetrance tables derived by Culverhouse et al. (2002) and Wongseree et al. (2009). On the other hand, the minor allele frequencies (MAFs) of the remaining SNPs are between 0.05 and 0.5; these conform to the MAFs of SNPs targeted by the International HapMap Project (The International HapMap Consortium 2005). The allele frequency setting is similar to that in the early study by Wongseree et al. (2009). The data set consists of balanced case-control samples of size 1,600. All SNPs in control samples are in Hardy-Weinberg equilibrium. The case samples are drawn from two independent groups of affected individuals where the disease status of each individual from the same group is the result of the same purely epistatic interaction. This leads to the presence of genetic heterogeneity. The interesting ratios of case samples from two affected groups are 1:1 and 1:3. The genotype distribution of causative SNPs that produce an independent interaction follows the purely epistatic model, leading to the heritability of 0.01. Thirty independent data sets for each simulation setting are generated by genomeSIM (Dudek et al. 2006). Since the same simulated data sets are used during the benchmarking, a paired *t*-test can be applied to assess the significance of difference in algorithm performance.

**Figure 1:**
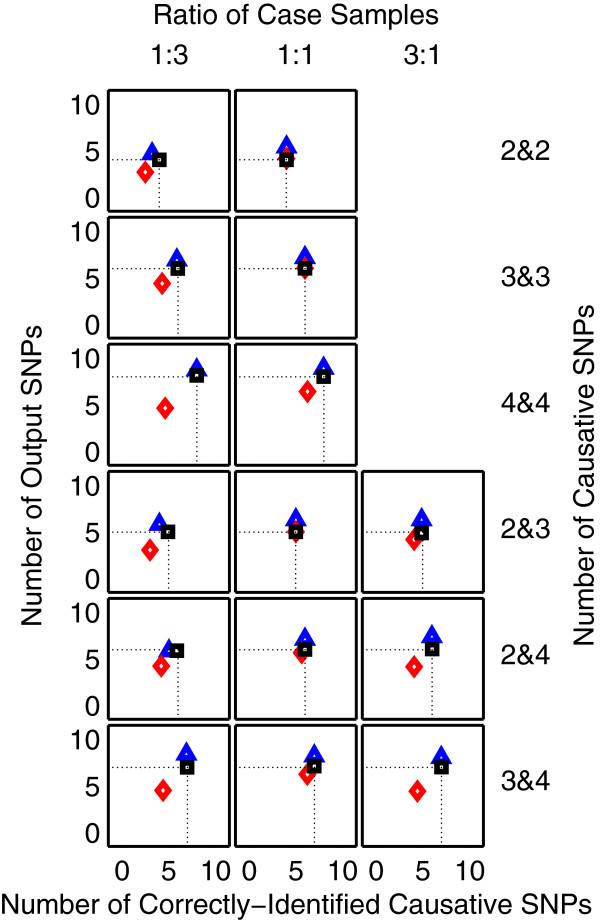
**Performance of MDR, RF and 2LOmb in the problem with 20 SNPs.** The results are averaged over 30 independent simulations. MDR explores only models that do not contain more than 10 SNPs. The MDR output contains the most parsimonious SNP combination that yields the maximum prediction accuracy. The number of trees in RF is set to 100. The RF output consists of top-ranked SNPs, which are SNPs with variable importance in the top five percentiles of a normal distribution (Strobl et al. 2009). Association detection is declared for 2LOmb if the global *p*-value used as the detection indicator in its result is less than 0.05. The results from MDR, RF and 2LOmb are displayed using red diamond, blue triangle and black square markers, respectively. In each chart, the meeting point between two dotted lines denotes the graphical location representing ideal performance of the algorithm. Ideally, the algorithm should report only the causative SNPs in its output. In other words, both number of output SNPs and number of correctly-identified causative SNPs should be equal to the number of causative SNPs. The charts on which the red diamond markers are invisible denote the situations in which the performance of MDR and 2LOmb is similar.

**Figure 2:**
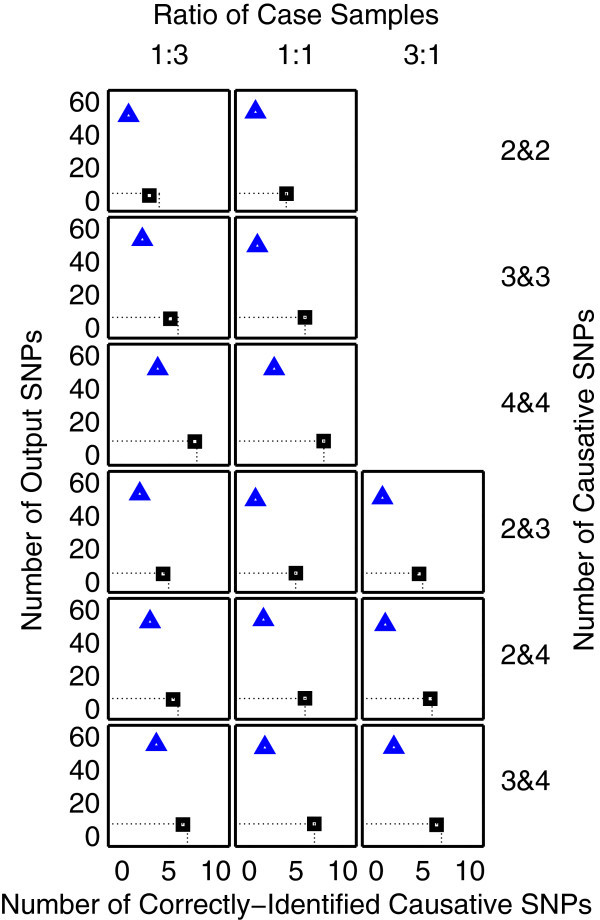
**Performance of RF and 2LOmb in the problem with 1,000 SNPs.** The number of trees in RF is set to 1,000. The explanation for how the results are obtained and displayed is the same as that given in Figure 1. The charts in this figure are displayed using a coarser scale than the charts in Figure 1.

The results from the problems with 20 and 1,000 SNPs in data are shown in Figures 1 and 2, respectively. It can be seen from Figure 1 that MDR fails to detect some causative SNPs. This suggests that the detection capability of MDR is lower than that of both RF and 2LOmb. Since it is highly unlikely that the MDR performance can improve when the number of available SNPs increases, the MDR simulation for the problem with 1,000 SNPs is not carried out. As a result, the MDR simulation is limited to the problem with 20 SNPs, which is similar to the study by Edwards et al. (2009). In Figures 1 and 2, the parameter setting of 1:3 for the ratio of case samples from two affected groups leads to two sets of results if the numbers of causative SNPs responsible for the two independent interactions are not equal. The first set of results is obtained when the low-order interaction is responsible for the affected status of individuals from the small-proportion group. On the other hand, the second set of results is obtained when the low-order interaction is responsible for the affected status of individuals from the large-proportion group. 2LOmb significantly outperforms MDR and RF in terms of the low number of output SNPs, the high number of correctly-identified causative SNPs or both in the problems with 20 and 1,000 SNPs (a paired *t*-test on 15×30=450 benchmark results for each problem yields a *p*-value < 0.05). The statistical power analysis also reveals that the benchmark trial with 30 independent data sets for each simulation setting is sufficient for an accurate evaluation of the overall algorithm performance (power > 0.95 for a type I error rate of 0.05). The simulation results can be further interpreted as follows.

MDR functions by attempting to identify a SNP combination which leads to the maximum prediction accuracy. In the presence of genetic heterogeneity, multiple SNP combinations are required where each combination is needed for the correct class prediction of a portion of case-control samples. If the proportions of samples at which their class labels can be predicted by different SNP combinations are equal, then each causative SNP contributes equally towards achieving high prediction accuracy. Subsequently, MDR is able to detect all or almost all causative SNPs. On the other hand, if the proportions of samples are not equal, then the attained prediction accuracy depends more on the ability to classify samples that occupy the large proportion. In other words, the inclusion of SNP combination necessary for the identification of class labels of samples that occupy the small proportion does not lead to an improvement of prediction accuracy. As a result, MDR fails to uncover some causative SNPs when the ratio of case samples from two affected groups is 1:3.

RF identifies causative SNPs by permuting the genotype of the interesting SNP and monitoring how it affects the prediction accuracy. It is aimed that the reduction in prediction accuracy as a result of the genotypic change of the causative SNP is more prominent than that of other SNPs. Although this is an efficient strategy, RF also selects erroneous SNPs as causative SNPs. This is observed from the number of output SNPs reported by RF which is greater than the number of correctly-identified causative SNPs. The number of erroneous SNPs increases drastically when the number of available SNPs increases from 20 to 1,000. Moreover, the number of correctly-identified causative SNPs also markedly decreases. This can be explained from the manner at which each tree is constructed. Basically, the tree construction begins by assigning a SNP, which provides the best split, from a randomly chosen SNP set as the root node, creating a split according to the genotype and sorting samples to the appropriate descendant node. This process is repeated until each final descendant node is assigned with samples from the same class or the maximum tree size, dictated by the number of available SNPs, is reached. Permuting the genotype of a SNP located at or near the root node produces a large effect on the prediction accuracy while permuting the genotype of a SNP in a descendant node produces a small effect. Since the chance that a causative SNP being located near or at a root node is small when the problem contains a large number of SNPs, the variable importance of causative SNPs obtained by the genotype permutation may not be markedly different from that of other SNPs. This subsequently leads to the degradation of the RF performance. Although the performance of RF can be improved by increasing the number of trees in the forest (Strobl and Zeileis 2008), it also leads to an increase in computational time. The computational time result for RF, which will be discussed later, provides evidence against the option of increasing the number of trees for this study.

As mentioned earlier, 2LOmb produces the best results among three techniques in the benchmark trial. 2LOmb is capable of detecting most of causative SNPs in every simulated data set. This performance is further strengthened by highly significant *p*-values (2LOmb’s global *p*-value < 0.0001) and the presence of common SNPs among some or all SNP pairs that are parts of three- and four-locus interactions in the 2LOmb results. Nonetheless, some causative SNPs are missing from the 2LOmb output. Since the study is carried out by varying the number of available SNPs, the number of causative SNPs and the ratio of case samples from two affected groups, these parameters may influence the number of correctly-identified causative SNPs. The parameter analysis is divided into two parts. The first part concentrates on the results from problems where the numbers of causative SNPs responsible for two independent interactions are equal while the second part concentrates on those where the numbers of causative SNPs are not equal. The analysis is divided in this manner because as mentioned earlier the parameter setting of 1:3 for the ratio of case samples from two affected groups leads to two sets of results in only the second part of the analysis. From both parts, analysis of variance (ANOVA) reveals that all three parameters are the sources of variation which significantly affect the number of correctly-identified causative SNPs (*p*<0.05).

It is observed that the number of correctly-identified causative SNPs decreases when the number of available SNPs is large. This is to be expected because the Bonferroni correction factor is a quadratic function of the number of available SNPs. An increase in the Bonferroni correction factor leads to an increase in the Bonferroni-corrected *χ*^2^’s *p*-value. If there are not enough samples for the two-locus analysis to produce a sufficiently low Bonferroni-corrected *χ*^2^’s *p*-value, some causative SNPs may be excluded from the output ensemble. This is highly evident when the ratio of case samples from two affected groups is 1:3. The variation in the ratio of case samples also leads to a change in the number of correctly-identified causative SNPs. The magnitudes of Bonferroni-corrected *χ*^2^’s *p*-values for causative SNP pairs are similar when the numbers of case samples from two affected groups are equal. All causative SNPs can generally be identified in this scenario. On the other hand, the Bonferroni-corrected *χ*^2^’s *p*-values for SNP pairs responsible for the affected status of individuals from the small-proportion group are higher than those for SNP pairs responsible for the affected status of individuals from the large-proportion group. As a result, the exclusion of causative SNP pairs with insufficiently low Bonferroni-corrected *χ*^2^’s *p*-values from the output ensemble leads to a decrease in the number of correctly-identified causative SNPs.

In contrast to the first two parameters, an increase in the number of causative SNPs leads to an increase in the number of correctly-identified causative SNPs. This phenomenon can be explained as follows. To identify a multi-locus interaction, a number of SNP pairs must be included in the output ensemble. For instance, an ensemble of three SNP pairs namely (SNP1, SNP2), (SNP2, SNP3) and (SNP1, SNP3) leads to the identification of a three-locus interaction between SNP1, SNP2 and SNP3. However, only two-out-of-three SNP pairs are necessary for the correct interaction identification. Similarly, only three-out-of-six possible SNP pairs are necessary for the correct identification of a four-locus interaction. In other words, the number of redundant SNP pairs increases as the order of interaction increases. Hence, the number of correctly-identified causative SNPs increases when there are more redundant SNP pairs, which can be omitted from the output ensemble.

In addition to the superiority in terms of the number of output SNPs and the number of correctly-identified causative SNPs, the computational time for 2LOmb analysis is tractable. The order of growth in 2LOmb computational time is *O*(*mn*^2^) where *m* is the sample size and *n* is the number of available SNPs. The computational time for RF analysis is also tractable. However, the order of growth in RF computational time is $O(m\log(m)\sqrt{n}f)$ where *f* is the number of trees, signifying that the required computational time also depends on the algorithm setting (Guyon and Elisseef 2006). In contrast, the tractability of MDR depends on the maximum size of explored models. If MDR explores all possible SNP combinations, the order of growth in MDR computational time is *O*(*m*2^*n*^), which makes the computational time becomes intractable. On the other hand, if MDR explores only models that do not contain more than *n*_*s*_ SNPs where *n*_*s*_<*n*, the order of growth in MDR computational time is $O(mn^{n_{s}})$, which means that the computational time is tractable. Since the computational time required by 2LOmb, RF and MDR with the latter setting is all tractable, the comparison of computational time is hence carried out. MDR explores only models that do not cover more than 10 SNPs in the 20-SNP data sets. An MDR permutation test is also omitted because it requires large computational efforts and is only performed to assess the probability that the null hypothesis of no association is true. The summary of computational time required by all three techniques is given in Table 1. RF uses lesser computational time than 2LOmb while MDR uses more computational time than 2LOmb to analyse 20-SNP data sets. However, the computational time required by RF to analyse 1,000-SNP data sets is greater than that required by 2LOmb. In addition, by limiting the MDR analysis to the exploration of models that do not cover more than 10 SNPs, it is estimated using the present MDR result that the computational time required by MDR to analyse a 1,000-SNP data set is 2.75×10^21^ seconds. The present MDR result also suggests that the computational time required by MDR to perform a permutation test using 1,000 permutation replicates on a 20-SNP data set is 6.37×10^6^ seconds. The estimation of computational time conforms to the results from early reports (Edwards et al. 2009; Pattin and Moore 2008; Ritchie et al. 2001; Wongseree et al. 2009). This means that a direct application of MDR and RF used in this study (see Methods for details) to larger data sets in which all SNPs exhibit no marginal single-locus effects is certainly impractical. Overall, 2LOmb outperforms MDR and RF in this study. There are many attribute selection techniques that have been successfully applied to genetic association studies (Heidema et al. 2006; Motsinger et al. 2007; Van Steen 2012). It would be interesting to benchmark 2LOmb against other techniques that can also be applied to data containing pure epistasis (Culverhouse 2012; Jiang et al. 2011b; Zhang and Liu 2007) and genetic heterogeneity (Culverhouse 2012).

**Table 1:** Computational time required by MDR, RF and 2LOmb to analyse small-scaled simulated data sets with different numbers of available SNPs, different numbers of causative SNPs and different ratios of case samples from two affected groups

**Number of**	**Ratio**	**Computational time (sec)**				
**causative**	**of case**	**MDR**	**RF**		**2LOmb**	
**SNPs**	**samples**	**20 SNPs**	**20 SNPs**	**1,000 SNPs**	**20 SNPs**	**1,000 SNPs**
2&2	1:3	6,505	2	539	5	24
	1:1	6,434	2	529	6	23
3&3	1:3	6,573	2	529	13	32
	1:1	6,611	2	531	14	32
4&4	1:3	6,372	2	534	32	45
	1:1	6,528	2	538	27	46
2&3	1:3	6,637	2	529	12	32
	1:1	6,644	3	527	10	30
	3:1	6,776	2	528	10	28
2&4	1:3	6,513	2	525	16	35
	1:1	6,637	2	528	16	35
	3:1	6,599	2	528	18	34
3&4	1:3	6,369	2	526	22	38
	1:1	6,410	2	530	25	45
	3:1	6,435	2	528	22	38

The simulation is carried out on a computer server. The computer server is equipped with a Xeon 2.66 GHz quad-core processor and 4GB of main memory. A CentOS 5.5 operating system is installed on the computer server. The computational time is collected from the processing of multiple independent data sets for each simulation setting. The displayed time is the maximum time required by each algorithm to analyse one data set.

Another advantage of using 2LOmb for detecting pure epistasis in the presence of genetic heterogeneity is the ability to identify the number of independent interactions. This is possible because 2LOmb reports its output in the form of an ensemble of SNP pairs. If there are common SNPs between pairs, then the detection of a multi-locus interaction is declared. On the other hand, the absence of common SNPs between pairs signifies that the interactions are independent. For example, an ensemble that contains SNP pairs (SNP1, SNP2), (SNP3, SNP4) and (SNP4, SNP5) indicates the presence of genetic heterogeneity in which a two-locus interaction between SNP1 and SNP2 and a three-locus interaction between SNP3, SNP4 and SNP5 are independently responsible for the disease status of each individual. Obviously, it is impossible to directly identify the number of independent interactions from the MDR and RF results because both techniques report their outputs in the form of a set of SNPs and not a set of SNP pairs. To demonstrate this capability of 2LOmb, the previously described simulation is extended where the number of independent data sets for each simulation setting increases from 30 to 100. The portions of independent data sets in which 2LOmb can identify at least one interaction and both interactions in the data sets are obtained for the calculation of statistical power. Detection of one interaction is declared if 2LOmb correctly identifies at least two interacting causative SNPs responsible for the affected status of individuals from only one case group. On the other hand, detection of two interactions is declared if 2LOmb correctly identifies at least four interacting causative SNPs in the form of two SNP pairs without a common SNP among the pairs. In addition, each SNP pair must be responsible for the affected status of individuals from a different case group. The statistical power to detect genetic heterogeneity summarised in Table 2 indicates that 2LOmb can identify both interactions in nearly all 20-SNP data sets. However, a loss of statistical power to detect both interactions is observed when the number of available SNPs increases from 20 to 1,000. In particular, this occurs when the ratio of case samples from two affected groups is 1:3 and a two-locus interaction is responsible for the affected status of individuals from the small-proportion group. This conforms to the early observation regarding the effects of increasing the number of available SNPs and increasing the number of causative SNPs on the number of correctly-identified causative SNPs. In brief, the Bonferroni correction factor increases when the number of available SNPs increases. If the Bonferroni-corrected *χ*^2^’s *p*-value for a causative SNP pair is not low enough, this pair would be excluded from the output ensemble. Subsequently, a failure to identify the causative SNP pair that is solely responsible for the affected status of individuals from the small-proportion group leads to the reduction in statistical power to detect both interactions.

**Table 2:** Statistical power of 2LOmb to detect genetic heterogeneity in small-scaled simulated data sets with different numbers of available SNPs, different numbers of causative SNPs and different ratios of case samples from two affected groups

		**Statistical power**			
		**20 SNPs**		**1,000 SNPs**	
**Number of**	**Ratio**	**At least**	**Two**	**At least**	**Two**
**causative**	**of case**	**one interaction**	**interactions**	**one interaction**	**interactions**
**SNPs**	**samples**	**detected**	**detected**	**detected**	**detected**
2&2	1:3	1.00	0.95	1.00	0.55
	1:1	1.00	1.00	1.00	1.00
3&3	1:3	1.00	1.00	1.00	0.88
	1:1	1.00	1.00	1.00	1.00
4&4	1:3	1.00	1.00	1.00	1.00
	1:1	1.00	1.00	1.00	1.00
2&3	1:3	1.00	0.93	1.00	0.60
	1:1	1.00	1.00	1.00	1.00
	3:1	1.00	0.98	1.00	0.94
2&4	1:3	1.00	0.94	1.00	0.63
	1:1	1.00	1.00	1.00	1.00
	3:1	1.00	1.00	1.00	0.99
3&4	1:3	1.00	1.00	1.00	0.88
	1:1	1.00	1.00	1.00	1.00
	3:1	1.00	1.00	1.00	0.97

Each data set consists of balanced case-control samples of size 1,600. The results indicate that 2LOmb detects at least one interaction in every data set (global *p*-value < 0.0001).

### Testing with large-scaled simulated data

In this part of the study, each simulated data set contains 10,000 or 100,000 unlinked SNPs where two independent purely epistatic two-locus interactions are present. Only the setting of two independent two-locus interactions is considered because the early simulation results given in Table 2 indicate that this scenario is the most difficult one when the number of available SNPs is large. The allele frequencies of all causative SNPs are 0.5 while the MAFs of the remaining SNPs are between 0.05 and 0.5. The data set consists of balanced case-control samples of size 1,600, 3,200 or 6,400. All SNPs in control samples are in Hardy-Weinberg equilibrium. The case samples are drawn from two independent groups of affected individuals where the ratios of samples from two affected groups are 1:1 and 1:3. The genotype distribution of interacting causative SNPs follows the purely epistatic model which gives the heritability of 0.01. One hundred independent data sets for each simulation setting are generated by genomeSIM for the evaluation of statistical power to detect genetic heterogeneity.

The summary of statistical power to detect genetic heterogeneity in Table 3 indicates that the ability to detect both interactions is highest when the ratio of case samples from two affected groups is 1:1 and is lowest when the sample size is 1,600 and the ratio of case samples from two affected groups is 1:3. This conforms to the early observation where a similar phenomenon is detected when the number of available SNPs is 1,000. However, once the sample size is doubled and quadrupled, the ability to detect both interactions increases significantly. Both interactions can be detected in almost all data sets containing 10,000 or 100,000 SNPs and 3,200 samples while both interactions can be detected in all data sets containing 10,000 or 100,000 SNPs and 6,400 samples. Obviously, an increase in sample size causes an increase in the *χ*^2^ test statistic during the two-locus analysis of causative SNPs. This suggests that increasing the sample size leads to a lower Bonferroni-corrected *χ*^2^’s *p*-value for the SNP pair responsible for the affected status of individuals from the small-proportion group. Subsequently, the chance this SNP pair being included in the output ensemble increases.

**Table 3:** Statistical power of 2LOmb to detect genetic heterogeneity in large-scaled simulated data sets with different numbers of available SNPs, different sample sizes and different ratios of case samples from two affected groups where the affected status is governed by a two-locus interaction

		**Statistical power**			
		**10,000 SNPs**		**100,000 SNPs**	
	**Ratio**	**At least**	**Two**	**At least**	**Two**
**Sample**	**of case**	**one interaction**	**interactions**	**one interaction**	**interactions**
**size**	**samples**	**detected**	**detected**	**detected**	**detected**
1,600	1:3	1.00	0.30	1.00	0.13
	1:1	1.00	1.00	1.00	1.00
3,200	1:3	1.00	0.98	1.00	0.92
	1:1	1.00	1.00	1.00	1.00
6,400	1:3	1.00	1.00	1.00	1.00
	1:1	1.00	1.00	1.00	1.00

The results indicate that 2LOmb detects at least one interaction in every data set (global *p*-value < 0.0001).

Bonferroni correction during the two-locus analysis plays an important role in keeping the number of output SNPs reported by 2LOmb close to the number of causative SNPs (Wongseree et al. 2009). However, the overly conservative nature of Bonferroni correction when the number of statistical tests is large (Jiang et al. 2011a) also leads to the aforementioned limitation in 2LOmb’s ability to detect both independent interactions when the ratio of case of samples from two affected groups is 1:3. Although increasing the sample size is a possible solution, other multiple testing correction techniques can be used instead of the Bonferroni correction to tackle this problem. For instance, false discovery rate (FDR) analysis is a strong candidate and is proven to be appropriate for DNA microarray data analysis (Storey and Tibshirani 2003) and genome-wide association studies (The Diabetes Genetics Replication and Meta-analysis Consortium 2012). Further studies are required to determine the effect of replacing the Bonferroni correction in the two-locus analysis within 2LOmb with the FDR analysis.

The computational time summarised in Table 4 indicates that the computational time for the large-scaled simulation is a linear function of sample size. This is to be expected because the construction of a 2 × 9 contingency table for each two-locus analysis requires the assignment of samples to the appropriate cells in the table, which is a linear-time operation. On the other hand, the computational time is a quadratic function of the number of available SNPs. Since the basic operation of 2LOmb is the two-locus analysis, 2LOmb can tackle large-scaled problems with fixed sample size and varied number of SNPs in quadratic time (Wongseree et al. 2009). Overall, the result agrees with the order of growth in computational time discussed in the small-scaled simulation section. Based on the computational time given in Table 4, it is estimated that 2LOmb requires 3.14×10^5^ seconds (87.2 hours) of computational time to complete the analysis of a data set containing 500,000 SNPs and 6,400 samples. This suggests that the computational time of 2LOmb is tractable for genome-wide association studies.

**Table 4:** Computational time required by 2LOmb to analyse large-scaled simulated data sets with different numbers of available SNPs, different sample sizes and different ratios of case samples from two affected groups where the affected status is governed by a two-locus interaction

**Sample**	**Ratio of**	**Computational time (sec)**	
**size**	**case samples**	**10,000 SNPs**	**100,000 SNPs**
1,600	1:3	34	3,106
	1:1	34	3,116
3,200	1:3	68	6,227
	1:1	68	6,256
6,400	1:3	135	12,503
	1:1	136	12,560

The simulation is carried out on a computer system with a graphics processing unit. The parallelism of the graphics processing unit is exploited to speed up the computation. The computer system is equipped with an AMD 2.8 GHz quad-core processor, 4GB of main memory and an NVIDIA GeForce GTX 285 graphics processing unit. The graphics processing unit contains 240 streaming processors sharing 1GB of GDDR3 memory. Each streaming processor has a clock rate of 1.48 GHz. An Ubuntu 9.10 operating system is installed on the computer system. The computational time is collected from the processing of multiple independent data sets for each simulation setting. The displayed time is the maximum time required to analyse one data set.

### Analysis of type 1 diabetes mellitus data

The presence of pure epistasis and genetic heterogeneity in a T1D data set is identified using 2LOmb. The data set, which is collected and screened by the Wellcome Trust Case Control Consortium (WTCCC), consists of 1,963 case samples and 2,938 control samples. The case samples are collected from affected individuals in the UK while the control samples are the results of the merging between 1,458 samples from the UK blood services and 1,480 samples from the 1958 British birth cohort. The data set contains 469,557 SNPs, which are genotyped through the Affymetrix GeneChip 500K Mapping Array Set and pass the WTCCC quality control (The Wellcome Trust Case Control Consortium 2007). The SNP set is primarily reduced by screening for SNPs within or near genes (Herold et al. 2009; Ritchie 2011) according to NCBI build 36.3 (dbSNP b129) coordinates. SNPs that are near a gene are located within 2,000 bases upstream of the start site or 500 bases downstream of the termination site for transcription. The SNP set is further reduced by removing SNPs that exhibit marginal single-locus effects or have MAFs below 0.1. SNPs that the genotype distribution within control samples departs from Hardy-Weinberg equilibrium are also discarded. The final SNP set contains 95,991 SNPs with no marginal single-locus effects (uncorrected *χ*^2^’s *p*-value > 0.05) from 12,146 genes.

**Figure 3:**
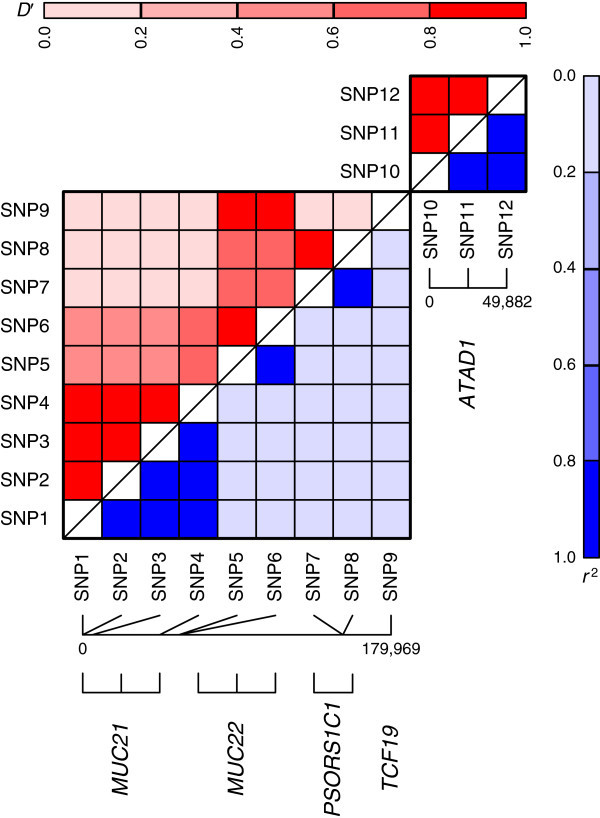
**LD patterns of SNPs within or near*MUC21*, *MUC22*, *PSORS1C1*, *TCF19 *and *ATAD1*.** LD is explained by *D*^′^ displayed in the upper triangle and *r*^2^ displayed in the lower triangle. Dark colours indicate high values while pale colours indicate low values. Distances between SNPs are given in terms of the number of base pairs. SNP1 = rs2844678, SNP2 = rs2523929, SNP3 = rs2530699, SNP4 = rs9262546, SNP5 = rs6933349, SNP6 = rs4713423, SNP7 = rs9263715, SNP8 = rs9263716, SNP9 = rs9263794, SNP10 = rs12775041, SNP11 = rs12573160 and SNP12 = rs12781171.

The analysis of the reduced T1D data set by 2LOmb takes 8,862 seconds (2.46 hours) of computational time on the computer system with a graphics processing unit (see Table 4 for detailed computer specification). The possible genetic association is detected from 12 SNPs located within or near five genes (global *p*-value < 0.0001). Details of these SNPs, the SNP pairs that exhibit marginal two-locus effects and the identified genes are given in Table 5. Linkage disequilibrium (LD) analysis is subsequently performed using JLIN (Carter et al. 2006) and the LD patterns are shown in Figure 3. All SNPs within or near the same gene are in LD due to high values of *D*^′^ and *r*^2^. This is most likely being the cause of the identification of multiple SNPs from the same gene. On the other hand, SNPs in each pair that contains SNPs from different genes (SNP pairs 2–18) are not in LD due to low values of *D*^′^ and *r*^2^. There are several subsets of these SNP pairs in which each subset contains three SNP pairs with common SNPs between pairs. One example is {(SNP1, SNP5), (SNP1, SNP7), (SNP1, SNP9)}. Consequently, the detection of these 17 SNP pairs indicates that there is a four-locus interaction between *MUC21* (mucin 21, cell surface associated), *MUC22* (mucin 22), *PSORS1C1* (psoriasis susceptibility 1 candidate 1) and *TCF19* (transcription factor 19). In contrast, there are no interactions between *ATAD1* (ATPase family, AAA domain containing 1) and the other genes due to the absence of a SNP pair containing a SNP from *ATAD1* and a SNP from any of the remaining four genes. The detection of three linked SNPs within *ATAD1* is believed to be the result of haplotype effects (Epstein and Satten 2003). Altogether, this clearly signifies the presence of pure epistasis and genetic heterogeneity. In real data analysis, the detection of a SNP pair that associates with the disease is insufficient to claim the presence of pure epistasis. If the SNP pair consists of two unlinked SNPs, then the detection of pure epistasis can be declared. Otherwise, the detection is the result of LD between SNPs. Since 2LOmb analysis cannot solely distinguish genetic association due to pure epistasis from genetic association due to LD, it is crucial to always perform additional LD analysis.

**Table 5:** 2LOmb identifies 12 SNPs, which are located within or near five genes, from the reduced T1D data set

	**Chromosome**	**SNP**		**SNP pair in the ensemble**																			
**Gene**	**and location**	**no.**	**SNP**	**1**	**2**	**3**	**4**	**5**	**6**	**7**	**8**	**9**	**10**	**11**	**12**	**13**	**14**	**15**	**16**	**17**	**18**	**19**	**20**
*MUC21*	6p21.32	1	rs2844678		●	●				●	●					●							
		2	rs2523929				●	●				●	●				●						
		3	rs2530699						●					●	●			●					
*MUC22*	6p21.33	4	rs9262546	●															●	●	●		
		5	rs6933349	●	●		●		●														
		6	rs4713423			●		●															
*PSORS1C1*	6p21.3	7	rs9263715							●		●		●					●				
		8	rs9263716								●		●		●					●			
*TCF19*	6p21.3	9	rs9263794													●	●	●			●		
*ATAD1*	10q23.31	10	rs12775041																			●	
		11	rs12573160																			●	●
		12	rs12781171																				●

Twenty SNP pairs are present in the ensemble. A pair of dots in the same column denotes a SNP pair.

The first four genes identified by 2LOmb, namely *MUC21*, *MUC22*, *PSORS1C1* and *TCF19*, are located on the major histocompatibility complex (MHC). MHC is a genomic region in which a mouse model of human complex diseases suggests the presence of T1D susceptibility genes (Cordell et al. 2001). The four genes are also located between *DDR1* (discoidin domain receptor tyrosine kinase 1) and *HLA-DQA1* (major histocompatibility complex, class II, DQ alpha 1), which is the region where the DR3-DQ2 ancestral haplotype 18.2 (AH18.2) is proven to be highly conserved and likely to carry susceptibility alleles for T1D in a Spanish population (Santiago et al. 2009). This implies that the detection of a four-locus interaction between these four genes conforms to the evidence from early genetic association studies of T1D. On the other hand, there are no early reports regarding the association between *ATAD1* polymorphisms and T1D. *ATAD1* is among many candidate genes for the association studies of Parkinson’s disease. Nonetheless, there is little information about pathways that include *ATAD1* (Moran and Graeber 2008). Hence, it is impossible to explain the association between *ATAD1* polymorphisms and T1D at this point.

This study produces evidence of association between 12 SNPs within or near *MUC21*, *MUC22*, *PSORS1C1*, *TCF19* and *ATAD1*, and T1D in a UK population. Although there are other independent genome-wide T1D data sets, the association detection within these data sets using the presented methodology has never been attempted. Basically, the methodology employed in most genome-wide association studies is based on single-locus analysis (Cooper et al. 2008; The Wellcome Trust Case Control Consortium 2007). Since each SNP explored in the reduced T1D data set exhibits no marginal single-locus effect, the most direct approach for replicating the association results presented in this article is to perform the same association detection on these independent data sets. This would certainly help to gain a further insight into the genetics of T1D.

## Conclusions

In this article, the detection of pure epistasis (Culverhouse et al. 2002) in the presence of genetic heterogeneity is investigated. The study focuses on the capability to detect two independent interactions that influence the development of the same complex disease. Each interaction can be either a purely epistatic two-locus interaction or a purely epistatic multi-locus interaction in which the causative SNPs exhibit no marginal single-locus effects. The candidate techniques for the detection benchmarking are MDR (Ritchie et al. 2001), RF (Breiman 2001) and 2LOmb (Wongseree et al. 2009). The results from various simulation scenarios indicate that 2LOmb outperforms MDR and RF in terms of a low number of output SNPs and a high number of correctly-identified causative SNPs. These scenarios are created by varying the number of available SNPs in data, the number of causative SNPs and the ratio of case samples from two affected groups. ANOVA reveals that all three simulation parameters influence the number of correctly-identified causative SNPs in the 2LOmb output. In addition to the superiority in the detection performance, 2LOmb is also capable of identifying the number of independent interactions. This is achieved through the identification of common SNPs among SNP pairs in the ensemble. The results indicate that 2LOmb is able to identify the presence of independent interactions even though the number of available SNPs reaches 100,000. Moreover, this is achieved in tractable computational time, which makes 2LOmb suitable for use in genome-wide association studies. 2LOmb is subsequently applied to a T1D data set, which contains 1,963 case samples and 2,938 control samples and is collected from a UK population (The Wellcome Trust Case Control Consortium 2007). The genome-wide data set is primarily screened for SNPs that locate within or near genes. The data set is further reduced by removing SNPs that exhibit marginal single-locus effects or have MAFs below 0.1. The final data set contains 95,991 SNPs from 12,146 genes. 2LOmb identifies 12 SNPs that are associated with the disease. These SNPs are located within or near *MUC21*, *MUC22*, *PSORS1C1*, *TCF19* and *ATAD1*. 2LOmb and LD analyses indicate that there is a four-locus interaction between *MUC21*, *MUC22*, *PSORS1C1* and *TCF19* while SNPs from *ATAD1* are independently associated with the disease. This signifies the presence of both pure epistasis and genetic heterogeneity. The evidence of genetic association for these five genes provides an alternative explanation for the aetiology of T1D in the UK population. It also confirms that SNPs which exhibit no marginal single-locus effects from a genome-wide data set can be useful for genetic association studies (Wongseree et al. 2009).

## Methods

### Purely epistatic model

A purely epistatic model is first defined by Culverhouse et al. (2002). The model describes an interaction between unlinked SNPs which leads to an epistatic effect while each interacting SNP exhibits no marginal single-locus effect. As a result, it is impossible to screen for SNPs contributing to pure epistasis by by single-locus *χ*^2^ tests for allelic and genotypic association. However, pure epistasis can be detected by multi-locus analysis. In this study, each model contains two, three or four causative SNPs. The purely epistatic three- and four-locus interaction models also exhibit marginal two-locus effects. All models yield the heritability of 0.01, which implies that genetic factors partially contribute towards disease susceptibility. The penetrance tables, which define the probability that an individual with a specific genotype has the disease, for purely epistatic two-, three- and four-locus interaction models used throughout the simulations are given in Tables 6, 7 and 8, respectively. Detailed derivation of these models is given in Culverhouse et al. (2002) and Wongseree et al. (2009).

**Table 6:** Two-locus penetrances that lead to the heritability of 0.01

	**Penetrance of genotype**		
**Genotype**	***BB***	***Bb***	***bb***
*AA*	0	0	4*K*
*Aa*	0	2*K*	0
*aa*	4*K*	0	0

*AA* and *BB* denote homozygous wild-type genotypes. *Aa* and *Bb* denote heterozygous genotypes. *aa* and *bb* denote homozygous variant genotypes. All allele frequencies are equal (*p*_*A*_ = *p*_*B*_ = 0.5). *K* = 1/201.

**Table 7:** Three-locus penetrances that lead to the heritability of 0.01

		**Penetrance of genotype**							
	***CC***			***Cc***			***cc***		
**Genotype**	***BB***	***Bb***	***bb***	***BB***	***Bb***	***bb***	***BB***	***Bb***	***bb***
*AA*	0	0	16*K*	0	0	0	0	0	0
*Aa*	0	0	0	0	4*K*	0	0	0	0
*aa*	0	0	0	0	0	0	16*K*	0	0

*AA*, *BB* and *CC* denote homozygous wild-type genotypes. *Aa*, *Bb* and *Cc* denote heterozygous genotypes. *aa*, *bb* and *cc* denote homozygous variant genotypes. All allele frequencies are equal (*p*_*A*_ = *p*_*B*_ = *p*_*C*_ = 0.5). *K* = 1/901.

**Table 8:** Four-locus penetrances that lead to the heritability of 0.01

			**Penetrance of genotype**							
		***CC***			***Cc***			***cc***		
**Genotype**		***DD***	***Dd***	***dd***	***DD***	***Dd***	***dd***	***DD***	***Dd***	***dd***
	*BB*	0	0	0	0	0	0	0	0	0
*AA*	*Bb*	0	0	0	0	0	0	0	0	0
	*bb*	0	0	64*K*	0	0	0	0	0	0
	*BB*	0	0	0	0	0	0	0	0	0
*Aa*	*Bb*	0	0	0	0	8*K*	0	0	0	0
	*bb*	0	0	0	0	0	0	0	0	0
	*BB*	0	0	0	0	0	0	64*K*	0	0
*aa*	*Bb*	0	0	0	0	0	0	0	0	0
	*bb*	0	0	0	0	0	0	0	0	0

*AA*, *BB*, *CC* and *DD* denote homozygous wild-type genotypes. *Aa*, *Bb*, *Cc* and *Dd* denote heterozygous genotypes. *aa*, *bb*, *cc* and *dd* denote homozygous variant genotypes. All allele frequencies are equal (*p*_*A*_ = *p*_*B*_ = *p*_*C*_ = *p*_*D*_ = 0.5). *K* = 1/3501.

### genomeSIM

genomeSIM is a software package for simulating case-control data in genetic association studies (Dudek et al. 2006). genomeSIM takes penetrance-based models as inputs necessary for dictating the case/control status of each sample. A case-control data set can be generated by a population-based simulation or a probability-based simulation. A population of genotype strings is initialised according to the allele frequency of each SNP in the population-based simulation. Successive generations are subsequently created through a forward-time simulation by crossing the genotype strings within each generation. This is pursued until the predefined number of generations is reached. On the other hand, genotype strings are incrementally created until the predefined numbers of case and control samples are obtained in the probability-based simulation. In this study, the probability-based simulation is employed to generate all case-control data sets. genomeSIM is available upon request to the Ritchie Lab, Center for System Genomics, Pennsylvania State University (Ritchie Lab 2013).

### Multifactor dimensionality reduction

MDR is a wrapper technique which is capable of identifying causative SNPs that are associated with a disease from case-control data (Ritchie et al. 2001). MDR functions by attempting to identify the best SNP combination that yields the highest prediction accuracy. The prediction accuracy is calculated by means of a 10-fold cross-validation. During the cross-validation, the data set is randomly divided into 10 folds of combined case-control samples in which 9 folds of samples are used to construct the prediction model while the remaining fold is used to test the model. The process of prediction model construction and testing is then repeated 10 times where for each time a different sample fold is chosen as the testing fold. The prediction model embedded in MDR is a multi-dimensional decision table with $3^{n_{c}}$ cells when *n*_*c*_ SNPs and all three possible genotypes according to each SNP are considered. Each cell in the decision table is filled with case and control samples for which their genotypes coincide with the cell labels. The ratio between the numbers of case and control samples dictates whether the genotype in each cell is a protective or disease-predisposing genotype. The prediction accuracy is then evaluated by counting the number of testing samples that their disease status can be correctly identified using the decision rules provided by the table.

Similar to other wrapper techniques, the total number of possible prediction models that MDR can explore is 2^*n*^−1 where *n* is the number of available SNPs in the data set. With the use of an exhaustive search, MDR can generally identify the best SNP combination that gives the highest prediction accuracy. However, the search for the best model can also be limited to models that do not cover more than *n*_*s*_ SNPs where *n*_*s*_<*n*. After exploring multiple prediction models with a fixed number of SNPs, MDR also returns an additional measure called cross-validation consistency. Basically, each time that a testing fold is used to determine the accuracy of the interesting prediction model, the attained accuracy can be compared with that from other models which have the same number of SNPs as the interesting model. The model with high cross-validation consistency is the one that consistently ranks the first in comparison to other models regardless of which testing fold being used. A model with high cross-validation consistency usually has high prediction accuracy. As a result, prediction accuracy remains the principal criterion for model selection while cross-validation consistency is only applied as an auxiliary criterion. If two or more SNP combinations give the highest prediction accuracy and have equally high cross-validation consistency, the most parsimonious combination—the combination with the least number of SNPs—is the one chosen as the best SNP combination.

A permutation test can subsequently be applied to estimate the probability that the null hypothesis of no association is true. Each permutation replicate is constructed by randomly assigning the case/control status to each sample with the constraint that the numbers of case and control samples must remain unchanged. MDR is then performed on each permutation replicate to obtain the best SNP combination together with its prediction accuracy and cross-validation consistency. The empirical *p*-value is given by the fraction of permutation replicates with the interesting measure larger than or equal to that obtained from the original data where the measure can be either prediction accuracy or cross-validation consistency (Hahn et al. 2003). MDR used in this study is publicly available from the Computational Genetics Laboratory, Dartmouth Medical School, Dartmouth College (Computational Genetics Laboratory at Dartmouth Medical School 2013).

### Random forest

RF refers to a collection or ensemble of decision trees (Breiman 2001). Each tree in RF is constructed in a top-down manner. The tree construction begins at the root node where an attribute (SNP) is selected as the test. Descendants of the root node are then created according to the values of this attribute (genotypes of this SNP). Next, the (case-control) data samples are sorted to the appropriate descendant node. The entire process is repeated using the samples associated with each descendant node to select another attribute to test at that point in the tree. This forms a forward search for an acceptable decision tree in which the search never backtracks to reconsider earlier node choices. Since there are multiple trees in the forest, RF takes a majority vote from the trees as the class decision. Hence, the trees should be diverse in order for the majority-vote concept to be applicable. It is suggested that an attribute for each node in a tree can be selected according to its suitability for being used as the test from a small group of randomly picked attributes. Empirical studies indicate that an attribute group size of $\left\lceil \right)\sqrt{\text{total number of attributes}}\rceil )$ is sufficient. Consequently, the samples allocated to each descendant node, which is created after selecting the most suitable attribute as the test, have lesser class variety. Moreover, each tree in RF is allowed to grow to its maximum size. This does not lead to data over-fitting because the overall class decision relies on outcomes from multiple trees in the forest.

Unlike MDR, a bootstrap aggregating or bagging approach provides a means to determine the prediction accuracy of RF. Given a (case-control) sample set, a bootstrap sample set with the size equals to original sample set is generated by sampling from the original sample set uniformly and with replacement. It is expected that 63.2% of bootstrap samples are unique while the remaining samples are duplicates. Original samples that are absent from the bootstrap sample set are referred to as out-of-bag samples. Bootstrap samples are employed during the tree construction while out-of-bag samples are used to evaluate the prediction accuracy. A new bootstrap sample set is generated for the construction of each tree. As a result, the votes are only counted across the trees that the sample is out-of-bag during the prediction accuracy evaluation. The application of a bootstrap aggregating approach also leads to a means to quantify attribute importance, which is commonly referred to as variable importance. The variable importance is measured using a permutation approach. By randomly permuting the value of the attribute of interest, the correlation between the attribute and the (case-control) class can be determined. When the permuted attribute and the remaining non-permuted attributes are used as inputs for RF to identify the class of out-of-bag samples, the prediction accuracy reduces markedly if the attribute of interest is correlated with the class. The average difference between the prediction accuracy obtained using the original attribute inputs and that obtained using the inputs with one permuted attribute over the trees is the variable importance. The standardised variable importance is defined as the quotient between the variable importance and a standard error derived from the between-tree variance of the variable importance. In other words, the standardised variable importance follows a standard normal distribution (Random Forests 2004). An attribute with variable importance in the top five percentiles of a normal distribution is considered to be in a top rank in comparison to other attributes and is hence correlated with the class. This decision criterion is similar to the one based on the extremity of variable importance suggested by Strobl et al. (2009). RF used in this study is publicly available from the Department of Statistics, University of California, Berkeley (Random Forests 2004). A review of RF for genetic association studies can be found in Goldstein et al. (2011). Interested readers should also refer to Schwarz et al. (2010) and Wei et al. (2013) for RF-based techniques that are computationally feasible for genome-wide association studies.

### Omnibus permutation test on ensembles of two-locus analyses

2LOmb is a filter technique which is specifically designed for detecting pure epistasis in case-control data (Wongseree et al. 2009). 2LOmb consists of four steps as follows.

#### Two-locus analysis

2LOmb begins by exhaustively performing two-locus analysis by *χ*^2^ tests. Each *χ*^2^ test determines the difference between the distribution of two-locus genotypes in case and control samples. For a case-control data set containing *n* SNPs, n2 two-locus analyses are attained. Subsequently, the *χ*^2^’s *p*-value from each two-locus analysis is adjusted by a Bonferroni correction. The Bonferroni-corrected *χ*^2^’s *p*-value from each two-locus analysis is min(n2× uncorrected *χ*^2^’s *p*-value, 1).

#### Permutation test

A permutation test is performed to test the null hypothesis $H_{0}^{e}$ that the ensemble *e* of two-locus analyses is not associated with the disease. To achieve this, a scalar statistic is first computed for the original case-control data set by combining Bonferroni-corrected *χ*^2^’s *p*-values for SNP pairs through a Fisher’s combining function ($-2\underset{i}{\sum }\log(p_{i})$). The calculation of the Fisher’s test statistic is then repeated for a set of permutation replicates. Each permutation replicate is constructed by randomly permuting the case/control status of each sample, which leads to different Bonferroni-corrected *χ*^2^’s *p*-values and Fisher’s test statistic. The *p*-value of the null hypothesis $H_{0}^{e}$ is then given by ?

(1) $$p_{0}^{e}=|\{i:1\leq i\leq t,T_{i}^{e}\geq T_{0}^{e}\}|/t$$

where $T_{i}^{e}$ is the Fisher’s test statistic calculated for the permutation replicate *i*, $T_{0}^{e}$ is the Fisher’s test statistic calculated for the original case-control data set, *t* is the number of permutation replicates and |·| denotes the size of a set.

#### Global *p*-value determination

Since multiple ensembles of two-locus analyses can be explored, the calculation of global *p*-value is required to adjust for multiple hypothesis testing. The result is the *p*-value of the global null hypothesis $H_{0}=\underset{1\leq e\leq E}{⋂}H_{0}^{e}$ in which none of *E* explored ensembles is associated with the disease. Similar to other omnibus permutation tests, the same set of permutation replicates that gives the raw or unadjusted *p*-value for each ensemble is also used to estimate the global *p*-value. To obtain the global *p*-value, the unadjusted *p*-value for the permutation replicate *i* of each hypothesis $H_{0}^{e}$ is first calculated from

(2) $$p_{i}^{e}=|\{j:0\leq j\leq t,j\neq i,T_{j}^{e}\geq T_{i}^{e}\}|/\mathrm{t.}$$

The *p*-value of the global null hypothesis *H*_0_ is then given by

(3) $$p_{\text{global}}=|\{i:1\leq i\leq t,p_{i}^{\min}\leq p_{0}^{\min}\}|/t$$

where $p_{i}^{\min}=\underset{e}{\min}p_{i}^{e}$ is the minimum of unadjusted *p*-values over the explored ensembles in the permutation replicate *i* and $p_{0}^{\min}=\underset{e}{\min}p_{0}^{e}$ is the minimum of raw *p*-values over the explored ensembles in the original case-control data set.

#### Search for the best ensemble of two-locus analyses

The search for the best ensemble of two-locus analyses initialises by selecting the SNP pair with the lowest Bonferroni-corrected *χ*^2^’s *p*-value, which is a part of result from the first step of algorithm. A permutation test is then performed for this two-locus analysis, yielding both raw and global *p*-values because only one hypothesis has been explored. If the raw and global *p*-values of this first ensemble are statistically insignificant, the search terminates and the null hypothesis of no association cannot be rejected. Otherwise, the search continues by merging the SNP pair with the next lowest Bonferroni-corrected *χ*^2^’s *p*-value to the current best ensemble and re-evaluating the raw and global *p*-values. The search continues progressively in this manner until either an increase in the raw or global *p*-value is observed or all possible SNP pairs are included in the ensemble. If the search terminates prior to the inclusion of all possible SNP pairs, the best ensemble is the one from the previous iteration.

In this study, the significance level (*α*) to determine whether an ensemble is associated with the disease is 0.05 and the number of permutation replicates is 10,000, which is proven to be sufficient in the early study (Wongseree et al. 2009). 2LOmb is publicly available from its homepage (Detecting Purely Epistatic Multi-locus Interactions by an Omnibus Permutation Test on Ensembles of Two-locus Analyses 2009).

### Java LINkage disequilibrium plotter

A Java LINkage disequilibrium plotter (JLIN) is a software package for the illustration of linkage disequilibrium patterns (Carter et al. 2006). JLIN is used to display *D*^′^ and *r*^2^ calculated for SNPs which are associated with T1D. JLIN is publicly available from the Centre for Genetic Epidemiology and Biostatistics, University of Western Australia (JLIN 2010).

## Abbreviations

2LOmb: Omnibus permutation test on ensembles of two-locus analyses; AH: Ancestral haplotype; ANOVA: Analysis of variance; ATAD1: ATPase family, AAA domain containing 1; dbSNP: Single Nucleotide Polymorphism Database; DDR1: Discoidin domain receptor tyrosine kinase 1; DNA: Deoxyribonucleic acid; FDR: False discovery rate; genomeSIM: Simulation package for generating case-control samples in genetic association studies; GYS2: Glycogen synthase 2 (liver); HLA-DQA1: Major histocompatibility complex, class II, DQ alpha 1; JLIN: Java LINkage disequilibrium plotter; LD: Linkage disequilibrium; LMX1A: LIM homeobox transcription factor 1, alpha; MAF: Minor allele frequency; MDR: Multifactor dimensionality reduction; MHC: Major histocompatibility complex; MUC21: Mucin 21, cell surface associated; MUC22: Mucin 22; NCBI: National Center for Biotechnology Information; PARK2: Parkinson protein 2, E3 ubiquitin protein ligase (parkin); PGM1: Phosphoglucomutase 1; PSORS1C1: Psoriasis susceptibility 1 candidate 1; RF: Random forest; SNP: Single nucleotide polymorphism; T1D: Type 1 diabetes mellitus; T2D: Type 2 diabetes mellitus; TCF19: Transcription factor 19.

## Competing interests

The authors declare that they have no competing interests.

## Authors’ contributions

DS performed the small-scaled simulations, large-scaled simulations and statistical analysis. PT performed the small-scaled simulations and monitored the execution of computer programs on the computer server. NJ performed the large-scaled simulations, analysed the T1D data and monitored the execution of 2LOmb on the computer system with a graphics processing unit. SK performed the small-scaled simulations and summarised the results. WW performed the statistical analysis and provided comments about genetic association studies. TP performed the statistical analysis and provided comments about experimental design. TU performed the small-scaled simulations and commented on the results. CL provided additional comments about the genetic association study of T1D. CA provided comments about the manuscript. MP assisted in parallelising 2LOmb and handling large-scaled data. NC conducted the literature survey, formulated the research question, designed the experiment, discussed all results, drew the conclusions and wrote the manuscript. All authors read and approved the final manuscript.

## Authors’ information

DS is a Ph.D. student at the Department of Electrical and Computer Engineering, Faculty of Engineering, King Mongkut’s University of Technology North Bangkok. He also received his B.Eng. degree in computer engineering from King Mongkut’s University of Technology North Bangkok. His research interests include machine learning, bioinformatics and genetic epidemiology.

PT received his B.Eng. degree in computer engineering from King Mongkut’s University of Technology North Bangkok. His research interests include machine learning and genetic epidemiology.

NJ is a software developer at the Department of Computer Engineering, Faculty of Engineering, King Mongkut’s University of Technology Thonburi. He received his B.Eng. degree in computer engineering and M.Eng. degree in electrical engineering from King Mongkut’s University of Technology North Bangkok. His research interests include high performance computing and bioinformatics.

SK received his B.Eng. degree in computer engineering from King Mongkut’s University of Technology North Bangkok. His research interests include machine learning and genetic epidemiology.

WW is a lecturer at the Division of Technology of Information System Management, Faculty of Engineering, Mahidol University. He received his B.Eng., M.Eng. and Ph.D. degrees in electrical engineering from King Mongkut’s University of Technology North Bangkok. His research interests include machine learning, evolutionary computation and bioinformatics.

TP is a post-doctoral researcher at the Department of Electrical and Computer Engineering, Faculty of Engineering, King Mongkut’s University of Technology North Bangkok. He also received his B.Eng. and M.Eng. degrees in production engineering as well as his Ph.D. degree in electrical engineering from King Mongkut’s University of Technology North Bangkok. His research interests include evolutionary multi-objective optimisation and machine learning.

TU received his B.Eng. and M.Eng. degrees in electrical engineering from King Mongkut’s University of Technology North Bangkok. His research interests include machine learning, bioinformatics and genetic epidemiology.

CL is the Head of Division of Molecular Genetics at the Department of Research and Development, Faculty of Medicine Siriraj Hospital, Mahidol University. He also received his M.D. degree from Mahidol University. His research interests include human genetics and genetic diseases.

CA is an assistant professor of computer science at the Department of Mathematics and Computer Science, Chulalongkorn University. He also received his B.Eng., M.Eng. and Ph.D. degrees in computer engineering from Chulalongkorn University. His research interests include machine learning, evolutionary computation and bioinformatics.

MP is an assistant professor of computer engineering at the Department of Computer Engineering, Faculty of Engineering, King Mongkut’s University of Technology Thonburi. He received his B.A. degree in electrical engineering from Brown University and received his M.Sc. and Ph.D. degrees in electrical and computer engineering from University of Wisconsin-Madison. His research interests include integrated circuit testing, fault tolerant systems, enterprise software development and high performance computing.

NC is an associate professor of electrical engineering at King Mongkut’s University of Technology North Bangkok and an adjunct professor of genetic epidemiology at Mahidol University. He received his B.Eng. and Ph.D. degrees from the Department of Automatic Control and Systems Engineering, University of Sheffield. His research interests include evolutionary computation, machine learning and genetic epidemiology.
